# Use of Virtual Surgical Planning in Oral Surgery: A Systematic Review

**DOI:** 10.7759/cureus.81051

**Published:** 2025-03-23

**Authors:** Mohammad Abrar Bhatt, Aashish Kemmu, Aakriti Choudhary, Ashwini Baghel, Bhuvaneshwari Parthasarathy, Aishwarrya P

**Affiliations:** 1 Oral and Maxillofacial Surgery, Armed Forces Hospital, Jazan, SAU; 2 Oral and Maxillofacial Surgery, HCG Cancer Center, Ahmedabad, IND; 3 Oral and Maxillofacial Surgery, SJAS Superspeciality Hospital, Dhanbad, IND; 4 Oral and Maxillofacial Surgery, Sri Aurobindo College of Dentistry, Indore, IND; 5 Oral and Maxillofacial Surgery, Meenakshi Ammal Dental College, Chennai, IND; 6 Orthodontics and Dentofacial Orthopedics, Dental College Azamgarh, Azamgarh, IND

**Keywords:** 3d imaging, craniomaxillofacial surgery, surgical accuracy, systematic review, virtual surgical planning

## Abstract

Virtual surgical planning (VSP) represents a significant advancement in craniomaxillofacial surgery by utilizing 3D imaging and computer-aided techniques to enhance precision and improve surgical outcomes. This research aimed to evaluate the effectiveness of VSP in comparison to traditional surgical planning methods. Following Preferred Reporting Items for Systematic Reviews and Meta-Analyses (PRISMA) guidelines, we conducted a systematic review of studies published between 2000 and 2024, sourced from MEDLINE/PubMed, Cochrane Library, and EMBASE. A total of 22 studies, including cohort, case-control, and randomized controlled trials, were analyzed to assess the impact of VSP on surgical accuracy, procedure duration, and patient outcomes. The findings revealed that VSP improves surgical accuracy and reduces operative times compared to conventional methods. However, challenges such as higher material costs and occasional inconsistencies in results remain. Despite these challenges, VSP holds significant potential to enhance precision and efficiency in oral surgery.

## Introduction and background

Advancements in medical technology have significantly impacted the field of surgery, leading to improvements in standardization, predictability, and overall outcomes. Among these innovations, virtual surgical planning (VSP) has emerged as a transformative approach, especially in craniomaxillofacial (CMF) surgery [1]. VSP combines advanced imaging and computer-assisted techniques to refine surgical planning, thereby improving the accuracy and efficiency of complex procedures [2,3].

Virtual surgical planning involves using three-dimensional (3D) imaging software to visualize and predefine surgical procedures. This preoperative planning method is especially important in craniomaxillofacial surgery, which addresses congenital and acquired conditions of the facial and cranial regions [3]. The facial anatomy, with its complex network of blood vessels, nerves, and vital structures like the brain, eyes, nose, and teeth, presents significant challenges in both surgical planning and execution [4]. VSP seeks to overcome these challenges by allowing surgeons to simulate and plan surgical procedures with greater precision.

The development of VSP dates back to the 1970s when computer-aided design/computer-aided manufacturing (CAD/CAM) technology started to standardize and predict surgical outcomes more effectively [5]. At the same time, computed tomography (CT) introduced the ability to capture human anatomy in three dimensions, laying the foundation for the development of computer-assisted surgical tools [6,7]. By the 1980s, the use of computer-assisted surgery in CMF procedures became more prominent, aided by advancements in Rapid Prototyping (RP) technology, which enables the rapid creation of scale models from 3D CAD data [8,9].

VSP comprises several key stages: data acquisition, CT image analysis, 3D anthropometric analysis, surgical simulation, and the design and production of patient-specific implants and surgical guides [10]. Tools like Romexis® CMF Surgery (Planmeca, Helsinki, Finland), ProPlan CMF® (Materialise, Leuven, Belgium), and Dolphin Imaging 3D Surgery® (Dolphin Imaging & Management Solutions, Los Angeles, CA, USA) facilitate comprehensive simulations of surgical steps, including skeletal structure osteotomies, post-oncological reconstruction, occlusion evaluation, and soft tissue responses to skeletal changes [11]. These tools improve surgical accuracy, reduce operating times, and enhance outcome predictability [12].

In craniomaxillofacial surgery, VSP has been particularly beneficial for procedures involving mandibular reconstruction, orthognathic surgery, maxillofacial trauma, and temporomandibular joint reconstruction [13]. The use of VSP tools such as 3D virtual planning, stereolithographic models, intraoperative cutting guides, dental splints, and patient-specific implants has significantly improved procedural accuracy and consistency [14]. Custom surgical guides made from materials like titanium, hydroxyapatite, or polyether ether ketone (PEEK) serve as a crucial link between virtual planning and the actual surgical procedure, reducing intraoperative uncertainty and improving precision [14].

Despite its benefits, the implementation of VSP faces challenges. High processing costs, extended delivery times, and the need for specialized training and coordination with external companies represent significant barriers [15]. Furthermore, the reliance on precise 3D integration with patient anatomical data, along with the potential for errors in segment identification, simulation, and guide fabrication, can affect surgical outcomes [15]. These challenges emphasize the need for a thorough understanding of VSP’s limitations and the importance of ongoing research to address these issues effectively.

Recent studies on VSP have highlighted both its potential and its limitations. Although VSP has been shown to offer substantial improvements over traditional surgical planning methods, concerns such as high costs, preparation time, and the inflexibility of patient-specific implants during intraoperative changes remain [16]. The current literature reveals a need for more comprehensive studies, particularly those with larger sample sizes and deeper insights from users skilled in modern medical technologies [17].

This systematic review aims to offer a thorough examination of the existing literature on VSP in craniomaxillofacial surgery. By synthesizing current research, this review seeks to enhance our understanding of VSP’s applications, benefits, and limitations. Additionally, it aims to provide recommendations for future research to address the gaps identified in previous studies and to further the development of VSP in oral surgery.

## Review

Materials and methodology

This systematic review has been conducted with full adherence to the Preferred Reporting Items for Systematic Reviews and Meta-Analyses (PRISMA) guidelines. A thorough methodology was developed in advance of the review process, in line with the recommendations outlined in the Cochrane Handbook for Systematic Reviews of Interventions. The primary research question guiding this review is: "In craniomaxillofacial surgery, how does the implementation of Virtual Surgical Planning (VSP) compare to conventional surgical planning methods in terms of procedural accuracy, operational efficiency, and overall patient outcomes?"

The aim of this systematic review is to address this question by compiling, collecting, and analyzing all available studies on the use of VSP in oral and craniomaxillofacial surgery. This involves assessing the comparative effectiveness of VSP relative to traditional planning methods, with a focus on its impact on surgical precision, procedure duration, and patient outcomes. The review will identify existing gaps in the literature and provide recommendations for future research. Ultimately, it aims to optimize the application of VSP to enhance surgical practices and improve patient care.

Search Strategy

**Figure 1:**
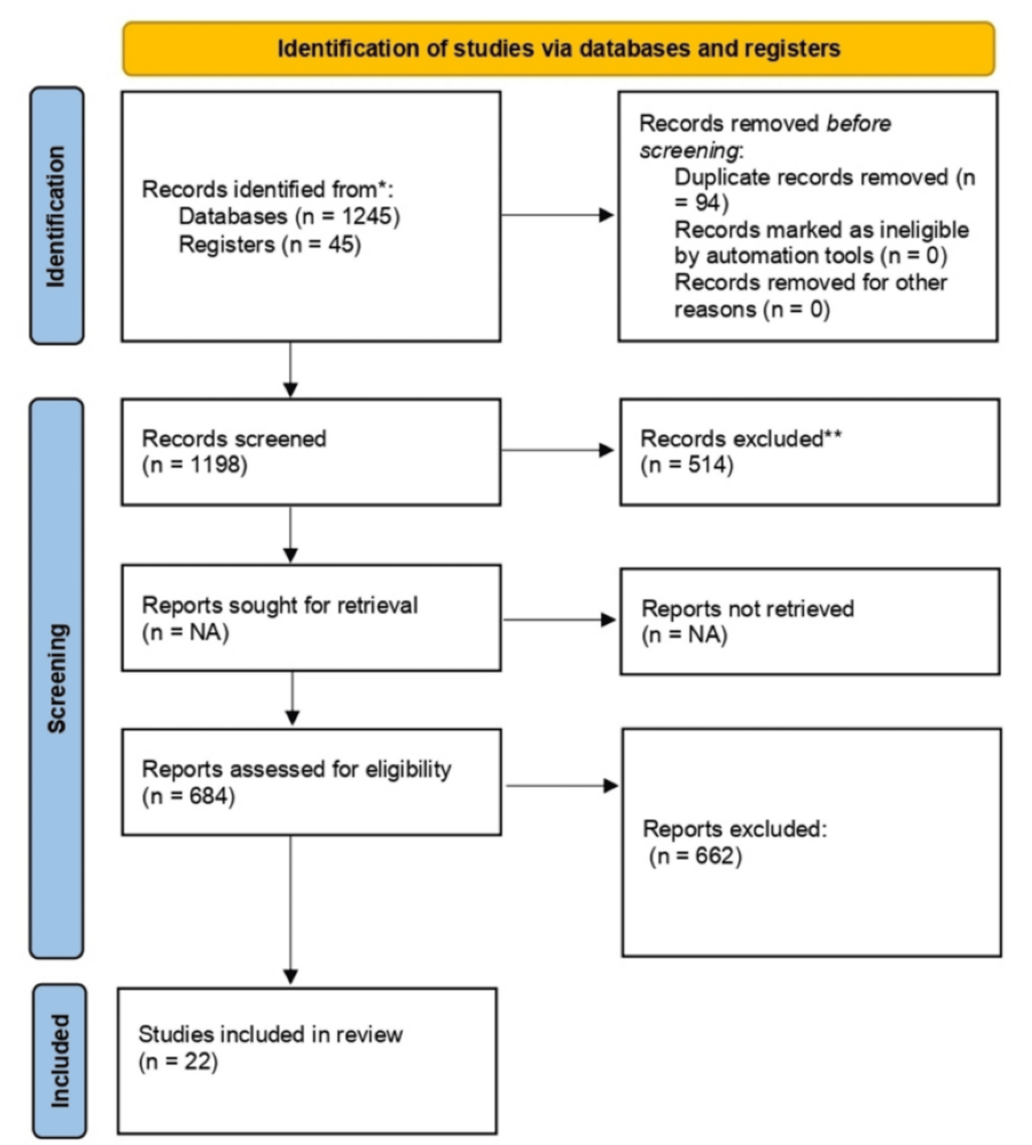
PRISMA Checklist PRISMA: Preferred Reporting Items for Systematic reviews and Meta-Analyses

A comprehensive search strategy was designed for undertaking an in-depth analysis of the evidence available regarding VSP in oral and craniomaxillofacial surgery. Our approach included an interdisciplinary methodology that integrates data from case-control studies, cohort studies, and randomized controlled trials (RCTs), hence covering a very wide spectrum of relevant research. Comprehensive literature searches were carried out in the key databases MEDLINE/PubMed, Cochrane Library, and EMBASE. We based our methodology on the PRISMA checklist to ensure rigorous standards of evidence, focusing on RCTs as the gold standard for quality data. Broad search terms were used, with no limits placed on language or date of publication, to maximize the coverage of relevant studies (Figure 1).

Literature was searched with relevant MeSH keywords that were carefully selected, coupled with specific keywords such as "Virtual Surgical Planning," "craniomaxillofacial surgery," "surgical outcomes," and "preoperative planning." A selective process was worked out with the aim of drawing out studies directly related to the review topic. The literature search included manuscripts published between 2000 and 2024 to capture very recent key studies. In addition to searching electronic databases, we conducted reference list scanning of the documents included in our review for other relevant articles that may not have emerged in the preliminary electronic database search.

This broad search strategy was chosen to construct a large body of evidence that would enhance present understanding of the application and current impact of VSP in oral and craniomaxillofacial surgery. Adherence to PRISMA guidelines and the use of a broad search strategy have increased this systematic review's validity and reliability. We also used PICOS (Population, Intervention, Comparison, Outcome and Study design) criteria to formulate the research question and decide the eligibility criteria for the studies included in this systematic review (Table 1).

**Table 1:** PICOS Criteria PICOS: Population, Intervention, Comparison, Outcome and Study design; VSP: virtual surgical planning; CAD/CAM: computer-aided design/computer-aided manufacturing

PICOS criteria	Explanation
POPULATION	Patients undergoing different oral and maxillofacial surgeries, such as tumor resections, mandibular reconstructions, and craniofacial advancements.
INTERVENTION	Use of VSP, 3D-printed surgical templates, CAD/CAM technology, and other advanced preoperative planning tools in oral and maxillofacial surgery.
COMPARISON	Comparison between VSP and traditional surgical planning methods or conventional techniques.
OUTCOME	Accuracy of surgical outcomes, intraoperative success, postoperative complications, adherence to surgical plans, operative time, and recovery metrics.
STUDY DESIGN	Cohort studies, case-control studies, and randomized controlled trials (RCTs).

Screening and Selection

Two reviewers selected the articles for this review through cooperative search and screening and arrived at a significant degree of agreement, as measured by a κ coefficient of 0.83. All the articles were reviewed in four stages in a disciplined manner. In Stage 1, irrelevant citations were quickly eliminated. Moving into Stage 2, one reviewer went through the abstracts and titles and eliminated items that were obviously beyond the boundary for inclusion. Uncertain circumstances underwent full-text screening and, when necessary, a second reviewer. At Stage 3, two reviewers independently assessed the material for confirmation that it met the qualifying criteria. Full review and data extraction of included publications have been made in Stage 4, focusing on details and the outcome of the intervention. This stringent procedure offers methodological robustness and relevance in line with the guidelines for medical research. A κ statistic calculated for the reliability of the screening process could further improve the validity of this review.

Data Extraction

The first author was involved in the first step of data extraction, while the second author was involved in the reviewing and refining. Presented in Table 2, identified articles with full text and meeting the inclusion criteria all had their data extracted in an independent fashion using a standardized format made possible through Microsoft Office Excel 2013 software (Microsoft, Redmond, WA, USA). Systematic sections were then created that covered such elements as authorship, year of publication, study design, participants, details about interventions, comparator elements, and outcomes. Because the methodology in this systematic review is very strict, high accuracy and consistency in extracting and formatting relevant data from each publication greatly enhance the overall strength of the data analysis.

**Table 2:** Data extraction sheet ANB: A point-Nasion-B point angle; CAD: Computer-aided design; CAD/CAM: Computer-aided design/computer-aided manufacturing; CSP: Conventional surgical planning; FFF: Fibula free flap; IS-VSP: Integrated surgical virtual surgical planning; LOS: Length of stay; MAO: Mandibular angle osteotomy; P-VSP: Patient-specific virtual surgical planning; PEEK-PSI: Polyether ether ketone patient-specific implants; SNA: Sella-Nasion-A point angle; SNB: Sella-Nasion-B point angle; TMJ: Temporomandibular joint; UHMWPE: Ultra-high molecular weight polyethylene; VSP: Virtual surgical planning.

Study	Population	Type of study	Mean age of patients	Parameters checked	Intervention	Comparison	Outcome	Time period
Efanov JI et al., 2018 [18]	54 patients	Retrospective review	Not specified	Adherence to virtual surgical plans (complete, partial, abandoned)	Virtual Surgical Planning (VSP)	Not specified	85% complete adherence, 9% partially adhered, 4% abandoned	July 2012 - October 2016
Yang WF et al., 2018 [19]	10 patients with head and neck tumors	Open-label, prospective, single-arm, single-center clinical trial	52.6 years	Intraoperative success rate, adaptation of surgical plates, postoperative adverse events, accuracy of reconstruction	3D-printed patient-specific surgical plates	Conventional plates (contingency plan)	Primary: 100% intraoperative success rate; Secondary: No major or minor complications; Mean absolute distance deviation: 1.40 ± 0.63 mm	December 2016 to October 2017
Sun et al., 2013 [20]	15 patients undergoing bimaxillary surgery (excluding cleft lip and palate)	Clinical validation study	Not provided	Accuracy of occlusal fit, surgical movement	Use of CAD-designed registration block (CAD_WR) and digital intermediate splint for bimaxillary surgery	Planned vs. Actual Surgical Movement	No significant difference between planned and actual surgical movements in sagittal, vertical, and horizontal directions	October 2010 to April 2012
Liu YF et al., 2014 [21]	15 cases (8 males, 7 females)	Clinical case series	39.8 years (range: 15–63 years)	Tumor type (ameloblastoma, fibroma, gingival carcinoma), defect size (3 cm × 3 cm - 10 cm × 5 cm), flap size (9.5 cm - 17 cm)	Fibular free flap reconstruction with template guidance	Conventional free-hand operation	Reduced surgical time by 20%, improved accuracy in resection and graft shaping, good postoperative functional recovery	December 2011 - December 2013
Soleman J et al., 2015 [22]	Infants with craniosynostosis	Observational study	8 months	Cranial morphology, symmetry, soft-tissue changes, operative outcomes	3D printed surgical templates for frontoorbital advancement	Standard procedures without 3D templates	Excellent accuracy in cranial modeling, improved reconstruction speed, minimal swelling, good scar healing, no neurological deficits	Postoperative follow-up: 3 weeks; Additional follow-up planned: 6 months
Ye N et al., 2015 [23]	Nine patients with prominent mandibular angles (8 women, 1 man)	Observational study	26 years	Accuracy of osteotomy, post-operative symmetry, deviation from simulation	Mandibular angle osteotomy (MAO) with 3D printed surgical templates	Standard MAO without 3D templates	High accuracy in osteotomy, minimal shell-to-shell deviations (2.02 ± 0.32 mm on right, 1.97 ± 0.41 mm on left), no nerve injury, good cosmetic outcomes	July 2013 to February 2014
Scolozzi P et al., 2015 [24]	Ten consecutive patients with dentofacial deformities (6 men, 4 women)	Retrospective study	21.3 years	Age, gender, dentofacial deformity, surgical procedure, postoperative complications	CAD/CAM surgical splints, cutting guides, customized internal distractors, and PEEK-PSI implants	Standard procedures without CAD/CAM	No intraoperative complications, stable cosmetic and dimensional results at 1-year follow-up	1 year follow up
Sembronio S et al., 2019 [25]	10 patients, 11 TMJ reconstructions (including bilateral)	Retrospective	Not specified	Prosthesis positioning accuracy, preoperative vs. postoperative measurements	Preoperative virtual planning and customized prostheses (titanium alloy and UHMWPE), surgical guides for bone resection and prosthesis placement	Preoperative vs. postoperative measurements	Lin concordance correlation coefficient: 0.999, 95% CI: 0.999–0.999, 99% CI: 0.999–1.000; 95% limits of agreement: -1.608 mm to 1.598 mm; 79.02% of differences <1 mm; no significant difference (p = 0.83)	2016-2017
Schneider D et al., 2019 [26]	21 patients with retrognathism	Prospective	31.1 years (median 32.6 years)	Angular differences for maxilla and mandible (SNA, SNB, ANB); accuracy of splints; surgical time; cost	Virtual Planning (VSP) vs. Conventional Planning (CSP)	Virtual Planning vs. Conventional Planning	Significant differences in angular measurements (SNA, SNB, ANB); fewer modifications needed for VSP splints; reduced surgical time with VSP; cost analysis showed VSP was similar to CSP without models	2014 to 2017
Smithers FAE et al., 2018 [27]	6 patients undergoing mandible or maxillary reconstruction	Prospective	Age range 44–78 years	Operative time, length of hospital stay, flap failures, wound infections, recovery complications	IS-VSP (Integrated Surgical Virtual Surgical Planning) process for fibula free flap (FFF) reconstructions	Conventional VSP	Median operative time: 7h 46min; Median length of stay: 13 days; No flap failures; One major complication (wound infection)	August 2016 to February 2017
Swendseid BP et al., 2020 [28]	23 patients with midface defects requiring scapula reconstruction	Retrospective cohort	Median age: 67 (range 22–88)	Subunit resection and reconstruction, anatomic position of bone segments, bone segment apposition, postoperative projection symmetry, shoulder dysfunction, quality of life	Virtual Surgical Planning (VSP) vs. non-VSP (conventional planning)	VSP vs. non-VSP	VSP group restored more subunits, achieved better anatomic positioning of bone segments, higher contact between bone segments, comparable operative times and quality of life	2015 to 2019
Sozzi D et al., 2022 [29]	21 patients (9 female, 12 male), mean age 45.9 ± 15.0 years (range 17–65)	Retrospective Study	45.9 ± 15.0 years	Pre-operative and post-operative positional accuracy of mandibular markers (e.g., condyles, midline, angles)	Virtual planning and surgical navigation, including hemi-mandibulectomy and free fibula flap reconstruction	Comparison of accuracy between right and left-hand sides	Overall discrepancy in mandibular positioning with higher error in mandibular angles; discrepancies analyzed by side	January 2010 – September 2018
Mazzola F et al., 2020 [30]	138 patients	Retrospective analysis	62.5 years (median)	Intensive monitoring days, ward length of stay, length of procedure, postoperative complications, bone segments used, screws, plates, ablative surgery details, donor site, complexity score, costs.	Non-VSP, P-VSP	Non-VSP vs P-VSP	P-VSP group had shorter median LOS, lower median ward costs, and operating costs despite higher material costs; no difference in complication rates.	January 2010 to March 2018
Kalmar CL et al., 2020 [31]	1131 consecutive craniofacial index procedures	Retrospective chart review	Varies by procedure	Gender, age at surgery, diagnosis, procedure type, VSP usage	Virtual Surgical Planning (VSP) vs. Traditional modalities	VSP vs. non-VSP	Increased VSP usage in certain procedures; variation in component utilization; trend towards higher VSP use over time	January 2011 - December 2018
Barrera JE et al., 2014 [32]	4 cases of obstructive sleep apnea (OSA) patients undergoing maxillomandibular advancement (MMA) surgery	Case series with analysis of surgical outcomes	40, 52, 48, 53 years	Airway measurements (PAS-O, PAS-M), AHI, RDI, LSAT, BMI, tooth-to-lip measurements	Virtual Surgical Planning (VSP)	Pre-surgical plan vs. Post-surgical results	Significant improvement in airway space, reduction in AHI and RDI, preservation of tooth-to-lip measurements, and maintenance of facial aesthetics	Follow up of 1 year
Qu X et al., 2017 [33]	52 patients undergoing mandibular reconstruction with osteocutaneous free flaps	Observational study	41.4 years (range 19–68)	Surgical accuracy (coincidence rate of fibular segments), implant placement accuracy, aesthetic outcomes	Virtual Surgical Planning (VSP) and double-barreled fibular reconstruction	Preoperative VSP vs. Postoperative outcomes	High coincidence rate for upper and lower barrels (93.43% and 89.72% respectively), significant improvement in ramus reconstruction, and generally positive aesthetic outcomes with a few issues	July 2010 – September 2016
Mendez BM et al., 2015 [34]	2 patients with complex craniofacial defects	Observational study	10 years (case 1), 34 years (case 2)	Cost and production time of 3D models, operative time, blood loss, hospital stay	In-office 3D printing technology for surgical models	None	Average cost of $25 and assembly time of 14 hours; successful surgeries with no perioperative complications	October 2014 – February 2015
Antonini F et al., 2020 [35]	100 adult patients who underwent two-jaw orthognathic surgery	Retrospective case–control study	22.1 years (range 14–46 years)	Accuracy of maxillary repositioning (x, y, z axes), overall accuracy, discrepancies between planned and postoperative results	Virtual Surgical Planning (VSP)	Different years of VSP execution (2013–2017)	Improved accuracy over years, with an increase in measurements within 1 mm discrepancy; decreasing discrepancies over time	March 2013 – September 2017
De Riu G et al., 2018 [36]	49 patients (19 males, 30 females)	Retrospective, Observational	26.4 years	15 angular and linear measures of jaw movements	3D planning with Maxilim® software, intermediate splints, bimaxillary orthognathic surgery	Maxilla-first vs. Mandibula-first, Genioplasty vs. No Genioplasty	Mean linear differences between planned and actual movements; significant differences in SNA, SNB, and anterior facial height	June 2011 to January 2016
Haq J et al., 2014 [37]	5 patients (3 females, 2 males)	Case series	44.6 years (range 29–58)	Maximum incisal opening; pain levels; heterotopic bone formation	Custom-made Biomet implants, virtual surgical planning, resection of ankylosis	Preoperative vs. Postoperative conditions	Improvement in mouth opening; recurrence of heterotopic bone in some cases	2010 to 2012
Li Y et al., 2015 [38]	12 patients (ages 18–35) from West China Hospital of Stomatology	Clinical study	Not explicitly stated	Linear differences, angular differences, precision of virtual planning	Computer-aided orthognathic surgery, virtual planning, guiding templates	Simulated vs. actual postoperative outcomes	Mean linear differences < 1.8 mm; Mean angular differences < 2.5 degrees; Accurate transfer of virtual plans to surgery	January 1 to August 31, 2014
Ying X et al., 2021 [39]	20 patients (16 women, 4 men)	Retrospective	25.00 ± 3.96 years	Landmark coordinates (x, y, z), RMSD, deviation analysis	Segmental LeFort I osteotomy, BSSRO, mandibular anterior subapical osteotomy, genioplasty	VSP vs. postoperative results	Acceptable accuracy of VSP, RMSD within clinical relevance	2018 to 2020

Risk of Bias Assessment

This systematic review utilized the ROBINS-I (Risk of Bias in Non-randomized Studies of Interventions) tool to assess the risk of bias (Table 3). This tool provides a framework for evaluating bias in estimates from non-randomized interventional studies. The risk of bias for each included study was carefully assessed across several domains. The first domain considered was confounding. For studies where relevant confounding variables or factors were not presented, the risk of bias was rated as higher. The next aspect examined was the potential bias arising from participant selection. This was assessed based on recruitment methods that could introduce systematic differences across comparison groups. Studies with inadequate selection procedures were deemed to have a higher risk of bias.

**Table 3:** Risk of Bias Assessment

Author	Bias due to confounding	Bias in selection of participants into the study	Bias in classification of interventions	Bias due to deviations from intended interventions	Bias due to missing data	Bias in measurement of outcomes	Bias in selection of the reported result	Overall bias
Efanov JI et al., 2018 [18]	Low	Low	Moderate	Low	Low	Moderate	Low	Low
Yang WF et al., 2018 [19]	Low	Moderate	Low	Low	Moderate	Low	Moderate	Moderate
Sun et al., 2013 [20]	Moderate	Low	Low	Low	Serious	Low	Low	Serious
Liu YF et al., 2014 [21]	Low	Low	Low	Low	Moderate	Low	Low	Low
Soleman J et al., 2015 [22]	Low	Low	Low	Low	Low	Low	Low	Low
Ye N et al., 2015 [23]	Moderate	Moderate	Low	Serious	Moderate	Low	Low	Serious
Scolozzi P et al., 2015 [24]	Low	Low	Moderate	Low	Low	Moderate	Low	Low
Sembronio S et al., 2019 [25]	Low	Low	Moderate	Low	Low	Moderate	Low	Low
Schneider D et al., 2019 [26]	Low	Moderate	Low	Low	Moderate	Low	Moderate	Moderate
Smithers FAE et al., 2018 [27]	Moderate	Low	Low	Low	Serious	Low	Low	Serious
Swendseid BP et al., 2020 [28]	Low	Low	Moderate	Low	Low	Moderate	Low	Low
Sozzi D et al., 2022 [29]	Low	Moderate	Low	Low	Moderate	Low	Moderate	Moderate
Mazzola F et al., 2020 [30]	Moderate	Low	Low	Low	Serious	Low	Low	Serious
Kalmar CL et al., 2020 [31]	Low	Low	Low	Low	Moderate	Low	Low	Low
Barrera JE et al., 2014 [32]	Low	Low	Low	Low	Low	Low	Low	Low
Qu X et al., 2017 [33]	Moderate	Moderate	Low	Serious	Moderate	Low	Low	Serious
Mendez BM et al., 2015 [34]	Low	Low	Moderate	Low	Low	Moderate	Low	Low
Antonini F et al., 2020 [35]	Moderate	Low	Low	Low	Serious	Low	Low	Serious
De Riu G et al., 2018 [36]	Low	Low	Moderate	Low	Low	Moderate	Low	Low
Haq J et al., 2014 [37]	Low	Moderate	Low	Low	Moderate	Low	Moderate	Moderate
Li Y et al., 2015 [38]	Low	Low	Moderate	Low	Low	Moderate	Low	Low
Ying X et al., 2021 [39]	Moderate	Low	Low	Low	Serious	Low	Low	Serious

Bias in the classification of interventions was also evaluated, focusing on whether the classification was consistent for all participants. Deviations from intended interventions were scrutinized for participant adherence, as well as how deviations were managed and reported. Studies with uncontrolled deviations were flagged for higher bias. Additionally, the handling of missing data and its potential impact on the results was assessed. Concerns were raised when outcome measurement tools were either inconsistent or unreliable, indicating potential bias. Finally, bias in the selection of outcome reporting was evaluated by determining whether all prespecified outcomes were reported. Studies that failed to report all of the prespecified outcomes were considered to have a higher risk of bias. The overall judgment about the methodological rigor and potential biases of the reviewed studies was derived by aggregating these assessments.

Results

Search and Selection

In the process of conducting this systematic review, 1,245 records were identified in the databases. There were 47 additional records from other sources, bringing the total to 1,292. After duplicates had been removed, there were 1,198 records remaining for screening. All the records were assessed, with 684 full-text articles screened for eligibility. In contrast, 514 records were excluded at the screening stage, and 662 full-text articles were excluded with stated reasons. Finally, 22 studies were included in this systematic review.

Analysis of Included Studies

This systematic review analyzed 22 studies with various designs aimed at evaluating the application of VSP in oral surgery. The studies included retrospective reviews, prospective trials, observational studies, clinical case series, and others. Efanov JI et al. (2018) conducted a retrospective review with 54 patients, revealing that 85% of virtual surgical plans were fully followed, 9% were partially followed, and 4% were abandoned, suggesting a strong integration of VSP into surgical planning from July 2012 to October 2016 [18]. In a similar vein, Yang WF et al. (2018) demonstrated a 100% success rate with minimal complications in intraoperative procedures using 3D-printed patient-specific osteosynthesis plates for tumors in the head and neck region, based on a prospective trial of 10 patients, outperforming traditional methods [19]. Sun et al. (2013) found no statistically significant differences between planned and actual surgical movements in bimaxillary surgeries using CAD-designed registration blocks and digital splints [20].

Liu YF et al. compared VSP-directed fibular free flap reconstructions with conventional methods, reporting 20% greater accuracy and reduced operative time [21]. A similar study by Soleman J et al. (2015) in infants with craniosynostosis found excellent accuracy in cranial modeling and surgical outcomes when using 3D-printed surgical templates [22]. High accuracy and low discrepancies in mandibular angle osteotomies using 3D-printed surgical templates were also noted in other studies [23], including retrospective and observational studies by Scolozzi P et al. (2015) and Sembronio S et al. (2019), which highlighted the benefits of CAD/CAM surgical splints in reducing intraoperative complications and improving prosthesis positioning accuracy [24,25]. Schneider D et al. (2019) observed significant improvements in angular measurements and reduced surgical times with VSP compared to traditional methods [26]. Smithers FAE et al. (2018) reported a median operative time of seven hours and 46 minutes using integrated surgical VSP (IS-VSP), with the procedure being much easier and less complicated [27].

Swendseid BP et al. (2020) identified advantages of VSP over conventional techniques in midface defects, including improved anatomical positioning and quality of life [28]. However, Sozzi D et al. (2022) found discrepancies in mandible positioning, particularly with greater errors at mandible angles [29]. Mazzola F et al. (2020) found that patient-specific virtual surgical planning (P-VSP) was linked to greater nutritional autonomy, better swallowing function, shorter hospitalization, and reduced overall costs, though material costs were higher [30]. Kalmar CL et al. (2020) observed a trend of increasing VSP use over the years, with variations in component utilization [31]. Barrera JE et al. noted significant postsurgical changes in airway space and facial aesthetics among patients with obstructive sleep apnea [32]. Furthermore, Qu X et al. (2017) affirmed that the accuracy of mandibular reconstructions using VSP and double-barreled fibular reconstructions was very high, while Mendez BM et al. highlighted cost-effective and successful outcomes using in-office 3D printing technology [33,34]. Antonini F et al. (2020) demonstrated that VSP increased accuracy and reduced discrepancies in orthognathic surgeries over the years [35]. De Riu G et al. (2018) found notable discrepancies in VSP accuracy regarding planned and actual jaw movements [36]. Haq J et al. (2014) concluded that the use of customized implants and VSP improved mouth opening [37]. Li Y et al. (2015) reported a mean linear measurement difference of less than 1.8 mm, indicating good accuracy in virtual planning for orthognathic surgeries. Lastly, Ying X et al. (2021) confirmed the clinical relevance of VSP, showing acceptable accuracy and minimal deviations in landmark coordinates [38,39].

Risk of Bias

**Figure 2:**
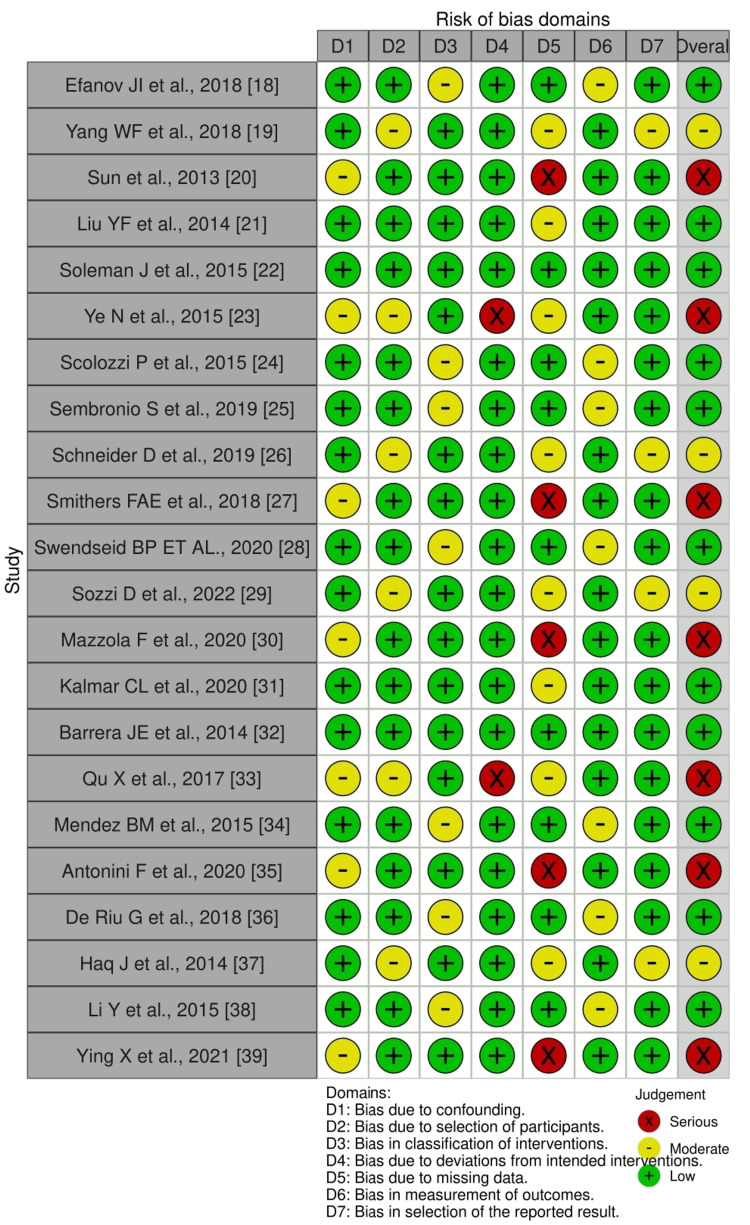
Summary plot

**Figure 3:**
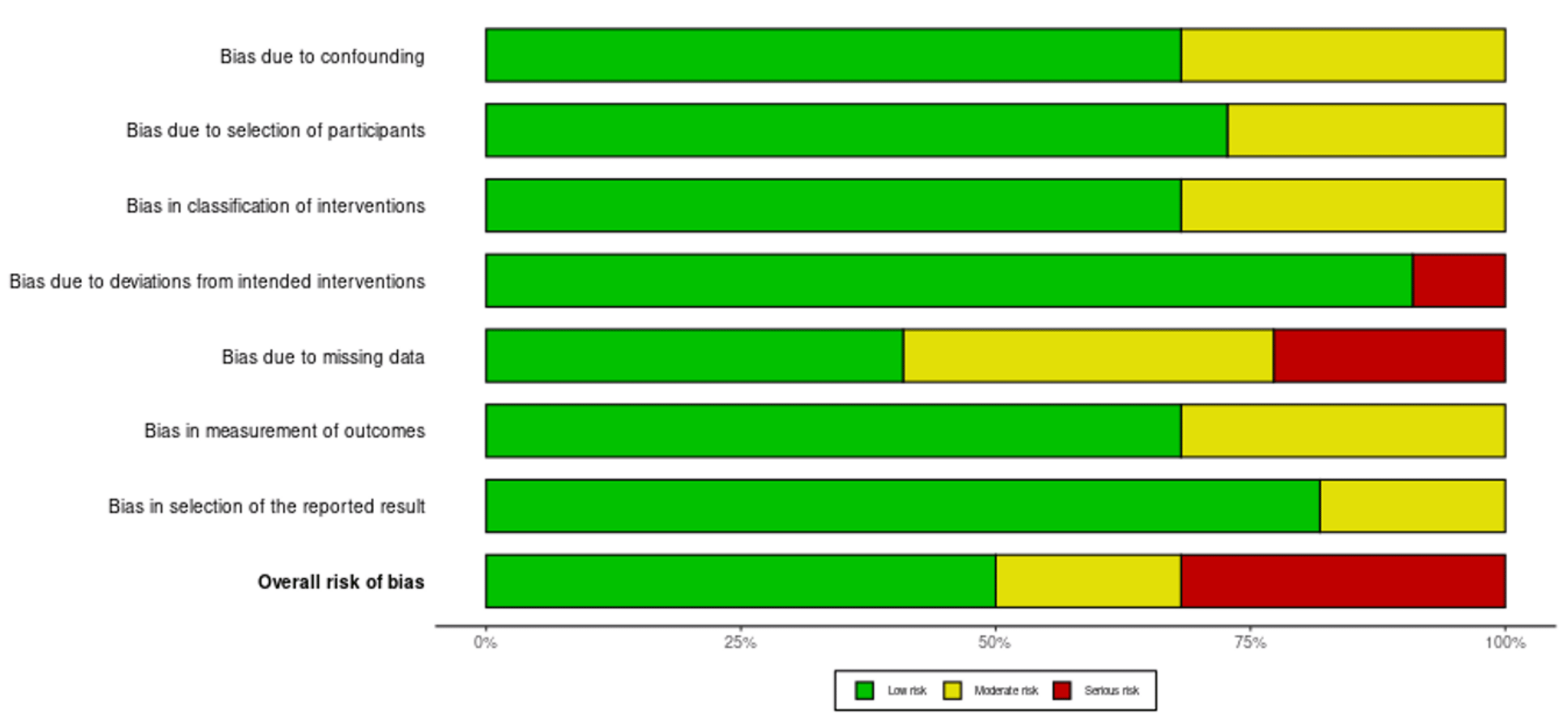
Traffic light plot

Several patterns of bias risk have been observed across the included studies (Figures 2, 3). Overall, most studies, including those by Efanov JI et al. (2018), Liu YF et al. (2014), and Soleman J et al. (2015), exhibited a low risk of bias due to confounding [18,22]. There was generally a low risk of bias in participant selection, although studies like those by Yang WF et al. (2018) and Ye N et al. (2015) showed a moderate risk. The classification of interventions ranged from low bias in the majority of studies to moderate bias in some, such as in the studies by Scolozzi P et al. (2015) [24] and Sembronio S et al. (2019) [25]. Bias related to deviations from intended interventions was mostly low, except in certain cases, such as in the studies by Yang WF et al. (2018) [19] and Qu X et al. (2017) [33], where deviations were more notable.

Bias due to missing data was generally considered low, but it became a significant concern in studies such as Sun et al. (2013) [22] and Mazzola F et al. (2020) [30]. Outcome measurement bias was typically low, although there were notable concerns regarding serious biases in studies like Smithers FAE et al. (2018) [27] and Ying X et al. (2021) [39]. While most results showed low bias, some studies, such as Schneider D et al. (2019) [26] and Sozzi D et al. (2022) [29], exhibited moderate bias. Consequently, the overall risk of bias varied across studies, with a considerable number of studies displaying significant bias, particularly in areas related to missing data and outcome measurement.

Discussion

The integration of VSP into oral surgery has been extensively explored in recent years, with several studies attempting to demonstrate its effectiveness in enhancing surgical outcomes. This systematic review compiles data from a range of studies to evaluate whether VSP truly impacts various surgical procedures and patient populations. The analysis indicates that VSP may serve as a catalyst for improving surgical precision, minimizing complications, and optimizing postoperative results.

Surgical Accuracy

VSP has shown a significant improvement in surgical accuracy across all types of procedures. Efanov JI et al. (2018) reported that 85% of cases adhered completely to the virtual surgical plan, emphasizing the role of VSP in ensuring high fidelity to preoperative plans [18]. Similarly, Schneider D et al. (2019) [26] found substantial differences in angular measurements when VSP was used, highlighting its ability to reduce variability in surgical outcomes compared to traditional methods. The results of Qu X et al. (2017) [33] on mandibular reconstructions further support the effectiveness of VSP, showing high coincidence rates for fibular segments and satisfactory aesthetic outcomes.

Comparison With Conventional Techniques

VSP generally offers significant advantages over conventional surgical techniques. Yang WF et al. (2018) [19] reported a 100% intraoperative success rate when 3D-printed patient-specific surgical plates were employed, outperforming traditional plates. Liu YF et al. (2014) [21] found that VSP-guided fibular free flap reconstructions reduced surgical time by 20% and improved accuracy compared to conventional free-hand techniques. These findings were further supported by Swendseid BP et al. (2020), who reported that VSP resulted in superior anatomical positioning and better bone segment contact in midface reconstructions, while maintaining equivalent operative times.

Outcomes and Complications

The impact of VSP on reducing postoperative complications is also highlighted in this review. Soleman J et al. (2015) reported excellent outcomes with 3D-printed surgical templates in infants with craniosynostosis, noting minimal swelling and good scar healing. However, studies such as Mazzola F et al. (2020) and Smithers FAE et al. (2018) indicated that while VSP reduces operative time and hospital stays, it does not significantly decrease complication rates like infection, excessive bleeding, dural tears, and postoperative cerebrospinal fluid leakage found in craniosynostosis surgery, when compared to non-VSP techniques.

Pre-operative Planning Accuracy

The success of VSP relies heavily on the accuracy of preoperative planning and its translation into the surgical field. Sun et al. (2013) and Li Y et al. (2015) reported minimal discrepancies between planned and actual outcomes, highlighting the precision of VSP in aligning preoperative plans with surgical execution [38]. However, Sozzi D et al. (2022) showed some mandibular positioning discrepancies, suggesting that ongoing refinement of VSP methods is necessary [29].

Economic Considerations

The economic aspects of VSP are a critical factor in its broader adoption. Mazzola F et al. (2020) [30] found that, despite higher material costs associated with VSP, there was a reduction in both hospital and operating costs. This finding is consistent with Antonini F et al. (2020), who showed that increased accuracy and reduced discrepancies over time could justify the investment in VSP through enhanced outcomes and efficiency [35].

Patient Demographics and Surgical Contexts

The effectiveness of VSP varies across different patient demographics and surgical contexts. Studies by Barrera JE et al. (2014) [34] and Haq J et al. (2014) [37] revealed significant improvements in airway space and functional outcomes, particularly in complex cases such as maxillomandibular advancement and ankylosis resection. These results highlight the benefits of VSP in challenging cases where precise planning and execution are essential.

Long-Term Outcomes and Follow-Up

Long-term follow-up data offers further insight into the sustainability of the benefits associated with VSP. Kalmar CL et al. (2020) [31] observed a trend toward increased VSP use over time, reflecting its growing acceptance and the long-term benefits that come with its integration into surgical practice. However, studies like De Riu G et al. (2018) [36] suggest that while VSP improves accuracy in the short term, further research is needed to fully understand its long-term advantages.

This systematic review, despite employing a rigorous methodology, has limitations. The heterogeneity of the included studies, the presence of variable biases, and the small number of high-quality randomized controlled trials (RCTs) limit the generalizability of the findings. The diverse applications and outcomes of VSP across studies also contribute to inconsistencies in the results. Additionally, reliance on studies published between 2000 and 2024 may overlook recent advancements and long-term effects of VSP. Further well-designed research is needed to address these gaps.

## Conclusions

This systematic review highlighted the notable advantages of VSP in oral surgery, including improved surgical precision, reduced operative times, and enhanced patient outcomes. VSP consistently outperformed traditional surgical methods, particularly in terms of accuracy and efficiency. However, the review also pointed to some challenges, such as the higher material costs and occasional inconsistencies in results, which suggest that further refinement of the VSP techniques is necessary. Additionally, long-term follow-up data showed promising results for VSP, indicating its potential for sustained positive effects. To fully realize its benefits, future studies should focus on addressing these challenges, optimizing the technology, and further evaluating VSP’s long-term impact on surgical outcomes and cost-effectiveness.

## Appendices

Acknowledgment
